# Gastrointestinal bleeding risk in cirrhotic portal vein thrombosis: focus on thrombus extension to superior mesenteric vein

**DOI:** 10.1186/s12876-026-04710-y

**Published:** 2026-03-03

**Authors:** Sa Lv, Hui Feng, Tianjiao Xu, Hua Tian, Haibo Wang, Dongze Li, Shaoli You, Bing Zhu

**Affiliations:** 1 Senior Department of Liver Diseases, Chinese PLA General Hospital, No. 100, Xisi Huanzhong Road, Beijing, 100039 China; 2 Department of Ultrasound, The Fifth Medical Center of Chinese PLA General Hospital, No. 100, Xisi Huanzhong Road, Beijing, 100039 China

**Keywords:** Portal vein thrombosis, Thrombosis classification, Gastrointestinal bleeding, Superior mesenteric vein thrombosis

## Abstract

**Background & aims:**

Portal vein thrombosis (PVT) is a prevalent cirrhosis complication linked to gastrointestinal bleeding. This study aims to assess correlations between PVT classification and the risk of bleeding in patients with cirrhotic PVT.

**Methods:**

This retrospective study included 380 hospitalized cirrhotic patients complicated by PVT, categorized into bleeding and non-bleeding groups based on history of gastrointestinal bleeding. Patients were followed for 12 months to calculate bleeding and recurrence rates. PVT was classified as non-extending or extending, based on the anatomical location and extent. Multivariate logistic regression analyses and propensity score matching (PSM) were used to evaluate the impact of superior mesenteric vein thrombosis (SMVT) extension on bleeding outcomes.

**Results:**

Of 380 patients, 223 (58.7%) were in the bleeding group and 157 (41.3%) in the non-bleeding group; 267 (70.3%) had non-extending PVT and 113 (29.7%) extending PVT (89 involving SMV). During follow-up, 201 (55.5%) experienced bleeding, with a 63.7% recurrence rate in the initial bleeding group. Extended PVT and SMVT-positive patients were significantly more likely to have higher baseline bleeding rates, 12-month bleeding rates, and recurrence rates compared with non-extended and SMVT-negative patients (all *P* < 0.05). Multivariate analysis identified SMVT extension as an independent risk factor associated with baseline (OR = 2.194; *P* = 0.010) and 12-month bleeding (OR = 1.962; *P* = 0.018). PSM confirmed significant associations between SMVT extension and gastrointestinal bleeding at baseline (*P* = 0.035) and 12 months (*P* = 0.033).

**Conclusions:**

In patients with cirrhosis and PVT, the extension into the SMV is significantly associated with an increased risk of gastrointestinal bleeding.

**Supplementary Information:**

The online version contains supplementary material available at 10.1186/s12876-026-04710-y.

## Introduction

Portal vein thrombosis (PVT) is a clinically significant complication of cirrhosis, affecting 14%–25% of patients, with an annual incidence rate of 4.6%. It is closely associated with the progression of portal hypertension [1]. Research indicates that approximately 20%–40% of patients with PVT develop moderate-to-severe varices, with bleeding risks increasing by 2–3 times compared to those without PVT [2]. Previous studies have identified multiple risk factors for gastrointestinal bleeding in cirrhosis, including Child-Pugh class B or C, high model for end-stage liver disease (MELD) scores, elevated D-dimer levels, low platelet counts, abnormal portal venous flow velocity, ascites, using non-selective beta-blockers (NSBBs), and severe esophageal varices. These findings suggest that hepatic decompensation and hemodynamic disturbances jointly contribute to increased bleeding [3, 4].

Recent advances have reshaped the therapeutic landscape for cirrhosis with PVT, shifting the clinical paradigm towards more proactive interventions, including anticoagulation and interventional radiology, as comprehensively reviewed by Wu et al. [5]. However, this evolution in treatment options underscores a critical challenge: the need for precise risk stratification to balance the competing risks of thrombosis progression and hemorrhage. Current risk assessment models, while valuable, often treat PVT as a homogeneous entity, potentially overlooking the prognostic significance of its anatomical heterogeneity.

Research on the risk factors of gastrointestinal bleeding in patients with cirrhosis complicated by PVT remains relatively scarce. Most existing studies have assessed bleeding risk by comparing the clinical variables between patients with and without PVT. This approach introduces potential confounding variables due to the inherent risk factors associated with PVT, thereby complicating the accurate determination of independent associations between PVT and bleeding risk [3]. Furthermore, PVT exhibits considerable phenotypic heterogeneity, which is influenced by anatomical location, host factors, and severity of cirrhosis. Current research has not yet established clear correlations between these phenotypes and the risk of gastrointestinal bleeding, which impedes the development of personalized bleeding prevention strategies tailored to different thrombotic types [6]. For instance, although complete occlusive PVT exacerbates symptoms of portal hypertension and increases complications following transplantation, its direct impact on gastrointestinal bleeding has not been independently evaluated [7]. Moreover, substantial methodological heterogeneity is evident across studies owing to inconsistencies in the cohort design, patient inclusion criteria, thrombus classification, and treatment protocols. These discrepancies hinder cross-sectional comparisons and meta-analyses, with the reliability and generalizability of conclusions being further constrained by the limitations of small-sample studies [1].

Building on this foundation, the current study aimed to systematically investigate the risk factors associated with gastrointestinal bleeding in patients with PVT, using a comprehensive retrospective cohort analysis. Furthermore, we examined the relationship between the PVT classification and bleeding risk. The overarching objective is to provide robust, evidence-based insights to support the development of precision management strategies for PVT, based on its classification. Specifically, we focused on the clinical significance of thrombus extension to the superior mesenteric vein (SMV), aiming to identify a high-risk subgroup that could benefit from tailored management strategies.

## Patients and methods

This study adopted a retrospective cohort design to systematically collect clinical data from patients admitted to the Fifth Medical Center of the PLA General Hospital. These patients were initially diagnosed with liver cirrhosis complicated by PVT during hospitalization between January 2022 and December 2023. Inclusion criteria were as follows: (1) Age ≥ 18 years; (2) diagnosis of cirrhosis in accordance with established criteria; (3) ultrasonography-confirmed PVT, with no previous definitive diagnosis of PVT or associated medical records. Exclusion criteria were as follows: (1) Presence of concurrent malignant tumors (such as liver tumors); (2) history of surgical interventions, including splenectomy, splenic embolization, transjugular intrahepatic portosystemic shunt (TIPS), or liver transplantation; (3) prior anticoagulant therapy before enrollment; (4) diagnosis of acute or chronic pancreatitis, as confirmed by medical history, amylase/lipase assays, and imaging studies.

**Fig. 1:**
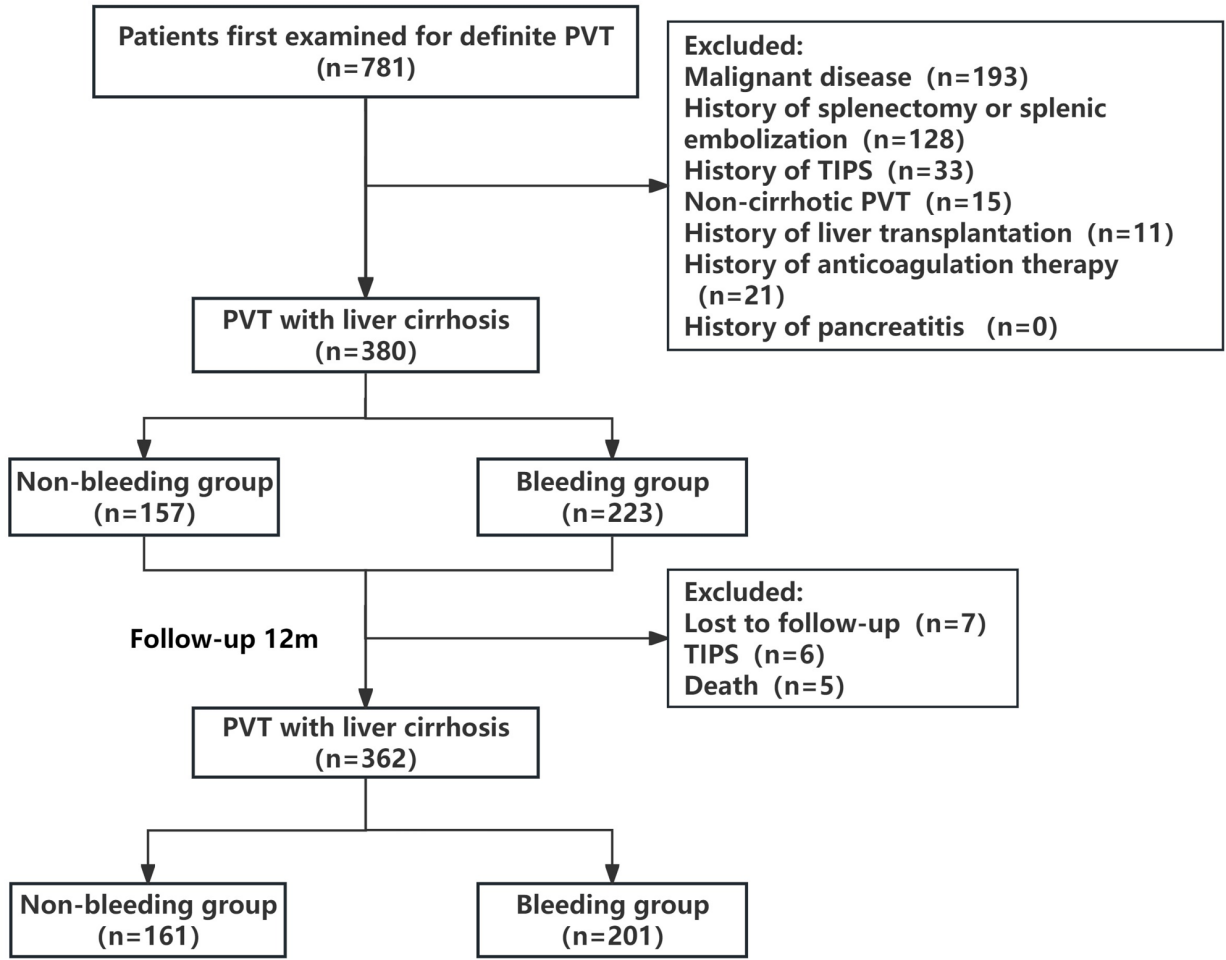
Study flowchart of patients with PVT and cirrhosis

Following a rigorous screening process, 380 patients were enrolled in this study. Participants were divided into bleeding and non-bleeding groups based on pre-enrollment gastrointestinal bleeding status, as shown in Fig. 1. This study was approved by the Ethics Committee of the Fifth Medical Center of PLA General Hospital (Approval No. KY20245741). Given the retrospective design of this study, the requirement for written informed consent was waived.

### Data collection

Upon patient admission, clinical data were systematically collected, including fundamental information, such as medical history, gender, age, etiology, and prior use of NSBBs. Laboratory test results, including complete blood counts, coagulation profiles, liver and kidney function tests, and relevant biochemical markers, were obtained. Hepatic function was assessed using the Child-Pugh and MELD scoring systems. The presence of esophageal or gastric varices was confirmed through gastroscopy, ascites was detected via ultrasound, and the severity of hepatic encephalopathy was evaluated according to the West Haven criteria [8]. Additionally, the anatomical location of PVT was documented based on ultrasound, computed tomography (CT), or magnetic resonance imaging (MRI) findings.

Gastrointestinal bleeding was defined based on clinical manifestations associated with gastrointestinal hemorrhage, such as hematemesis, coffee-ground vomiting, melena, or bloody stool, and was confirmed by endoscopy as esophagogastric variceal rupture bleeding. Patients in the bleeding group were defined as those who had the aforementioned bleeding manifestations either at the time of this hospitalization or prior to this admission. Severe esophageal varices were identified by the presence of esophageal veins exhibiting serpiginous tortuosity with redness or appearing as beaded, nodular, or tumor-like patterns, regardless of redness [9]. Moderate-to-severe ascites was defined as an abdominal fluid depth ≥ 3 cm, as determined by ultrasound examination [10].

### Classification criteria for PVT

According to the Sarin classification criteria [11], PVT in patients with cirrhosis was classified into two primary categories: Non-extended and extended. The non-extended category was characterized by thrombi confined to the main trunk of the portal vein (MPV) and/or branches of the portal vein (PV), which were further subdivided into three types: Type 1, in which the thrombus affected only the main trunk of the portal vein (MPV); Type 2, where it was restricted to the branches of the portal vein (PV branch); and Type 3, where both the main trunk and branches were involved (MPV + PV branch). Conversely, the extended category encompassed thrombi that extended to the splenic vein (SV) and/or superior mesenteric vein (SMV). This category was also divided into three subtypes: Type A thrombus extended solely to the SV, Type B thrombus extended only to the SMV, and Type C thrombus extended to both the SV and SMV.

### Follow-up

This study initiated follow-up from the date of the patients’ diagnosis of PVT, implementing a 12-month observation period, and concluding on December 31, 2024. The primary endpoint was gastrointestinal bleeding during follow-up. The secondary endpoint “rebleed” refers to recurrent gastrointestinal bleeding events in patients initially assigned to the bleeding group, which were confirmed as esophagogastric variceal rupture bleeding by endoscopy. Patients who did not develop primary endpoints but underwent TIPS procedures or liver transplantation, were lost to follow-up, or died were excluded from the final statistical analysis. The workflow of this study is shown in Fig. 1. For further analysis, patients were stratified into bleeding and non-bleeding groups based on the occurrence of bleeding during the 12-month follow-up period.

### Statistical analysis

All statistical analyses and associated chart plots were conducted using the R programming language (version 4.4.1). A two-tailed *P*-value of less than 0.05 was considered indicative of statistical significance. For continuous quantitative data, normally distributed variables were summarized as mean ± standard deviation (SD), and t-tests were used for intergroup comparisons. Non-normally distributed data were described using median values, and rank-sum tests were used for comparison. Categorical data were presented as frequencies (percentages), and intergroup comparisons were made using either the chi-square or Fisher’s exact test. Bivariate and multivariate logistic regression models were used to estimate the odds ratio (OR) and 95% confidence interval (CI) for differences in binary outcomes. The Kaplan-Meier method was used to construct Kaplan-Meier curves for the cumulative incidence of gastrointestinal bleeding. The Log-rank test was applied to compare differences in time to first bleeding between groups during follow-up, with a significance level α = 0.05. Variables with a *P*-value less than 0.1 in univariate logistic regression were included in the multivariate analyses. Propensity score matching (PSM) at a ratio of 1:2 was performed using the nearest neighbor method with a cutoff value of 0.1, facilitated by the MatchIt package. Adequate group balance was defined as achieving standardized mean difference (SMD) values below 0.2 following the matching process.

## Results

### Baseline characteristics of patients

The study recruited a cohort of 380 patients diagnosed with cirrhosis complicated by PVT, comprising 240 (63.2%) males and 140 (36.8%) females, with a mean age of 57.04 ± 10.10 years. Within this cohort, 157 patients (41.3%) were assigned to the non-bleeding group, and 223 patients (58.7%) were assigned to the bleeding group. The “Others” category of cirrhosis etiology (109 patients) comprised 58 patients with autoimmune liver disease, 15 patients with drug-induced cirrhosis, 22 patients with cryptogenic cirrhosis, 14 patients with metabolic-associated steatotic liver disease (MASLD), and 1 patient with Wilson’s disease. Notably, only four patients in the bleeding group had a history of NSBBs usage. A comprehensive overview of baseline characteristics, key laboratory parameters, complications, and thrombus distribution is shown in Table 1.

**Table 1:** Baseline Demographic, Biochemical, and Clinical Characteristics of 380 Patients with PVT

Variables	Non-bleeding Group (*n* = 157)	Bleeding Group (*n* = 223)	*P*-value
Demographics			
Male gender, n (%)	105 (66.9)	135 (60.5)	0.207
Age (years)	57.31 ± 11.34	56.86 ± 10.77	0.695
Etiology of cirrhosis, n (%)			0.589
- Viral	80 (51.0)	118 (52.9)	
- Alcohol	34 (21.7)	39 (17.5)	
- Others	43 (27.4)	66 (29.6)	
Child-Pugh Class, n (%)			0.392
- A	24 (15.3)	46 (20.6)	
- B	111 (70.7)	145 (65.0)	
- C	22 (14.0)	32 (14.3)	
Laboratory Parameters			
MELD score	7.60 (6.00, 10.70)	6.64 (6.00, 9.70)	0.065
AST (U/L)	36.00 (27.00, 48.00)	30.00 (22.00, 37.00)	**< 0.001**
ALP (U/L)	102.00 (74.00, 130.00)	86.00 (66.00, 122.00)	**0.046**
GGT (U/L)	34.00 (21.00, 69.00)	30.00 (18.00, 54.00)	**0.033**
TBIL (µmol/L)	26.40 (19.20, 40.30)	21.60 (15.30, 33.65)	**0.004**
TBA (µmol/L)	41.00 (23.00, 76.00)	24.00 (7.00, 47.00)	**< 0.001**
GLU (mmol/L)	5.10 (4.50, 5.90)	5.40 (4.70, 7.15)	**0.004**
CRE (µmol/L)	74.00 (64.00, 91.00)	72.00 (64.00, 84.00)	0.207
LYMPH (× 10^9^/L)	0.67(0.47, 0.95)	0.58 (0.41, 0.79)	**0.003**
RBC (× 10^12^/L)	3.58 ± 0.71	3.23 ± 0.68	**< 0.001**
HGB (g/L)	110.84 ± 23.01	91.52 ± 22.95	**< 0.001**
HCT	33.42 ± 6.17	28.77 ± 6.37	**< 0.001**
PLT (× 10^9^/L)	59.00 (41.00, 84.00)	60.00 (43.00, 82.00)	0.793
Complications			
Encephalopathy, n (%)	15 (9.6)	17 (7.6)	0.505
Ascites severity, n (%)			0.397
- None	26 (16.6)	34 (15.2)	
- Mild (< 3 cm)	54 (34.4)	92 (41.3)	
- Moderate/Severe (≥ 3 cm)	77 (49.0)	97 (43.5)	
Severe esophageal varices, n (%)	42 (26.8)	86 (38.6)	**0.016**
Gastric Varices, n (%)	77 (49.0)	146 (65.5)	**0.001**
Thrombus Characteristics			
Thrombus non-extensions, n (%)			0.640
- Type 1 (MPV)	54 (43.5)	70 (49.0)	
- Type 2 (PV branch)	20 (16.1)	19(13.3)	
- Type 3 (MPV + PV branch)	50 (40.4)	54 (37.7)	
Thrombus Extensions, n (%)			
- SV	15 (9.6)	36 (16.1)	0.064
- SMV	24 (15.3)	65 (29.1)	**0.002**
Portal Cavernoma, n (%)	26 (16.6)	33 (14.8)	0.640
Portosystemic Shunt, n (%)	43 (27.4)	61 (27.4)	0.994

Data Presentation: Continuous variables are expressed as mean ± SD or median (IQR) based on distribution

Categorical variables are presented as n (%)

*Abbreviations*: *MELD* model for end-stage liver disease, *AST* aspartate transaminase, *ALP* alkaline phosphatase, *GGT* gamma-glutamyl transferase, *TBIL* total bilirubin, *TBA* total bile acid, *GLU* glucose, *CRE* creatinine, *LYMPH* lymphocyte count, *RBC* red blood cell count, *HGB* hemoglobin, *HCT* hematocrit, *PLT* platelet count, *PVT* portal vein thrombosis, *SV* splenic vein, *SMV* superior mesenteric vein

Boldface values indicate statistically significant results (*P* < 0.05)

In terms of complications, the bleeding group demonstrated a significantly higher incidence of severe esophageal varices (38.6% versus 26.8%; *P* = 0.016) and gastric varices (65.5% versus 49.0%; *P* = 0.001) than the non-bleeding group. Conversely, no significant differences were found between the two groups in terms of other complications, such as hepatic encephalopathy and the severity of ascites. Although no statistically significant differences were observed between the groups regarding the non-extended thrombosis classification, portal cavernous transformation, or rates of spontaneous portosystemic shunts, the bleeding group demonstrated significantly higher rates of thrombosis extending to the SMV (29.1% versus 15.3%; *P* = 0.002). Additionally, an increasing trend was observed in thrombosis extending to the SV (16.1% versus 9.6%; *P* = 0.064).

### Characteristics of PVT

In this study, involving 380 patients diagnosed with PVT secondary to liver cirrhosis, the non-extended type was found to be the predominant variant, occurring in 267 (70.3%) patients, whereas the extended type was observed in 113 (29.7%) patients. Examination of the specific thrombus distribution further revealed that 331 (87.1%) patients exhibited involvement of the portal vein trunk, 51 (13.4%) patients had SV involvement, and 89 (23.4%) patients presented with SMV involvement (Table 2). Among them, 27 (7.1%) patients had concurrent involvement of both the SV and SMV, while 4 (1.1%) patients presented with isolated extrahepatic involvement of either the SV or SMV. Notably, among the 89 patients with thrombus extension to the SMV, 1 (1.1%) patient required emergency surgery due to intestinal necrosis associated with superior mesenteric vein thrombosis (SMVT).

**Table 2:** Thrombosis classification and proportion of cases in 380 patients with PVT

Thrombus Category	Subtype	Vascular Involvement	Thrombus Diagram†‡	Cases (*n* = 380)	Proportion
Non-extensions	Type 1	MPV		124	32.6%
	Type 2	PV branch		39	10.3%
	Type 3	MPV + PV branch		104	27.4%
Extensions	Type A	SV		24	6.3%
	Type B	SMV		62	16.3%
	Type C	SV + SMV		27	7.1%

*MPV* main portal vein, *PV* portal vein, *SV* splenic vein, *SMV* superior mesenteric vein

†: Vascular labels in Diagram: 1 = Left portal vein branch; 2 = Right portal vein branch; 3 = Main portal vein trunk; 4 = Superior mesenteric vein (SMV); 5 = Splenic vein (SV)

‡: Thrombus color coding: Red fill = Definite thrombus; Yellow fill = Possible thrombus

### The bleeding rate of different PVT types

**Fig. 2:**
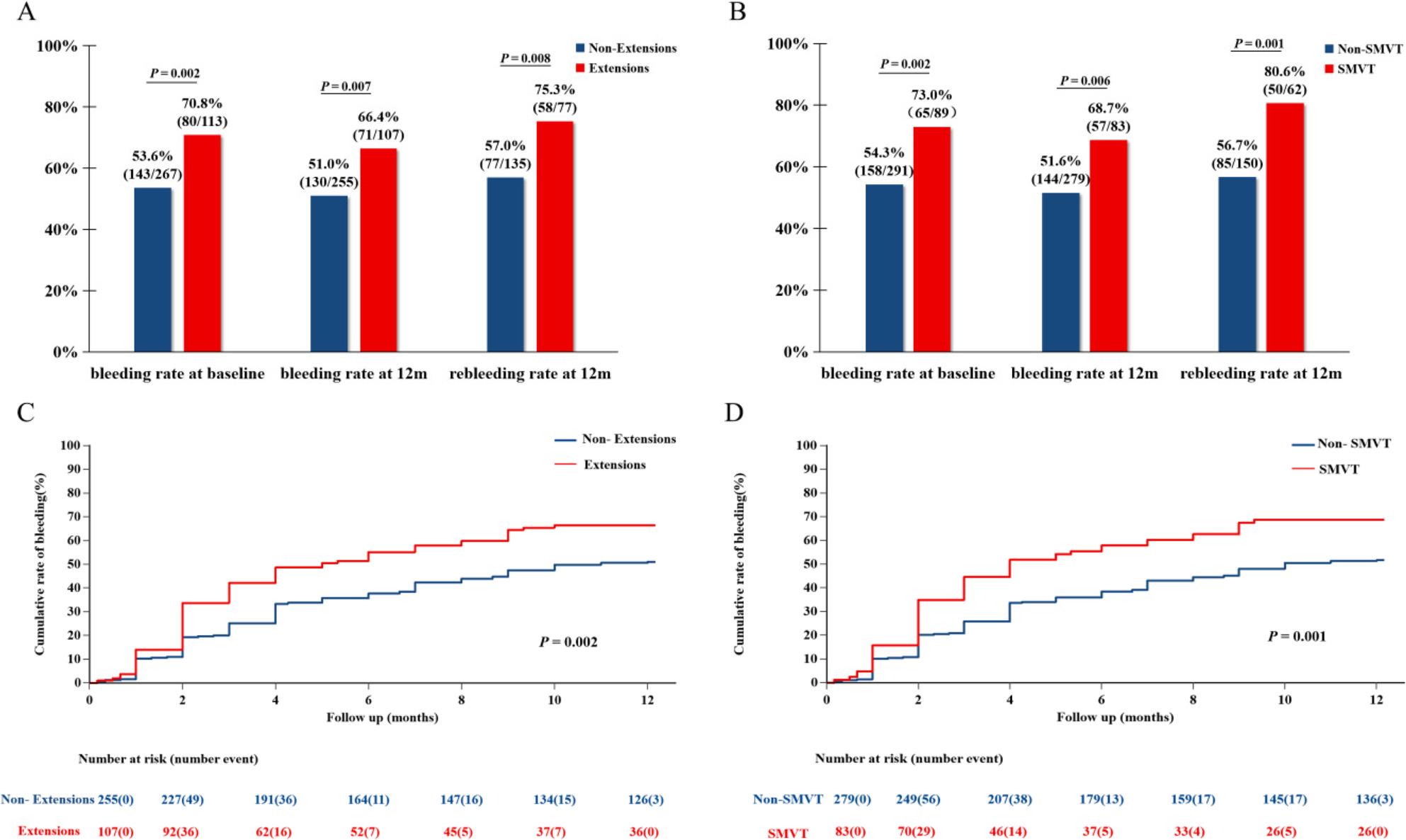
**A**. Bleeding, follow-up bleeding, and rebleeding rates at 12 months in patients with and without thrombus extension. **B**. Bleeding, follow-up bleeding, and rebleeding rates at 12 months in patients with and without SMVT extension. **C**. Kaplan-Meier curves for cumulative incidence of first gastrointestinal bleeding within 12-month follow-up with and without thrombus extension. **D**. Kaplan-Meier curves for cumulative incidence of first gastrointestinal bleeding within 12-month follow-up in patients with and without SMVT extension

In this study, a statistical analysis was conducted on the bleeding rates at enrollment among the different types of PVT. The findings revealed that the bleeding rate in patients with extended PVT was 70.8%, which was significantly higher compared to the 53.6% observed in patients without extension (*P* = 0.002) (Fig. 2A).

During the 12-month follow-up period, 362 of 380 patients successfully completed the follow-up for endpoint events, while 18 patients were excluded because of incomplete follow-up (7 in the non-bleeding group and 11 in the bleeding group, which included 6 extended-type cases and 12 non-extension-type cases). During the follow-up period, 201 (55.5%) patients experienced gastrointestinal bleeding or rebleeding. The rebleeding rate among the patients in the baseline bleeding group was 63.7% (135/212). Analysis further indicated that patients in the extended-type condition exhibited significantly higher rates of bleeding (66.4% versus 51.0%, *P* = 0.007) and rebleeding (75.3% versus 57.0%, *P* = 0.008) compared with the non-extension-type condition during the 12-month follow-up period (Fig. 2A).

The subgroup analysis stratified by the presence or absence of SMVT extension revealed that patients with SMVT extension experienced significantly higher bleeding rates at baseline enrollment (73.0% versus 54.3%, *P* = 0.002), during the 12-month follow-up period (68.7% versus 51.6%, *P* = 0.006), and rebleeding rates (80.6% versus 56.7%, *P* = 0.001) compared to those without SMVT extension (Fig. 2B).

To precisely delineate the temporal distribution of bleeding events, the Kaplan-Meier method was employed to generate curves depicting the cumulative incidence of initial gastrointestinal bleeding over a 12-month period (Fig. 2C and D). The findings indicated that the cumulative incidence of bleeding was significantly elevated in patients with extending PVT compared to those with non-extending PVT (*P* = 0.002). Similarly, patients who tested positive for SMVT exhibited a significantly higher cumulative incidence of bleeding than those who were SMVT-negative (*P* = 0.001). Furthermore, the cumulative incidence curves for both the extending PVT group and the SMVT-positive group demonstrated a more rapid increase during the initial 6 months of follow-up.

### Therapeutic interventions during follow-up

Throughout the follow-up period, all patients with viral etiology of cirrhosis received effective antiviral therapy, 35 patients (9.7%) were administered NSBB therapy, while 135 patients (37.3%) underwent endoscopic intervention. Anticoagulation therapy was provided to 8 patients (2.2%), including 2 patients with extension of SMVT. Of those receiving anticoagulation therapy, 4 patients (50%) experienced gastrointestinal bleeding during the follow-up, including both patients with SMVT extension (100%).

### Risk factors for baseline gastrointestinal bleeding in patients with PVT

This study incorporated 37 baseline variables and employed univariate logistic regression analysis to identify factors associated with the risk of gastrointestinal bleeding. The findings revealed that 10 variables were significantly associated with the risk of bleeding. The key indicators are shown in Table 3. Laboratory parameters, including AST, GGT, TBA, LYMPH, RBC, HGB, and HCT levels, significantly correlated with gastrointestinal bleeding. Among the factors related to varicose veins, the severity of esophageal varices (*P* = 0.017) and the presence of gastric fundus varices (*P* = 0.001) were also confirmed to be significantly associated with bleeding risk.

**Table 3:** Univariate and Multivariable Logistic Regression Analyses of Factors Associated with Gastrointestinal Bleeding in 380 Patients with PVT

Variable	Univariate Analysis		Multivariable Analysis	
	OR (95% CI)	*P*-value	OR (95% CI)	*P*-value
Sex (Female versus Male)	1.316 (0.860, 2.025)	0.208	–	–
Age	0.996 (0.978, 1.015)	0.691	–	–
Etiology of cirrhosis			–	–
Viral	Reference	–	–	–
Alcohol	0.778 (0.453, 1.338)	0.362	–	–
Others	1.041 (0.647, 1.683)	0.870	–	–
Child-Pugh class			–	–
B versus A	0.682 (0.388, 1.174)	0.173	–	–
C versus A	0.759 (0.363, 1.583)	0.461	–	–
MELD score	0.963 (0.911, 1.017)	0.173	–	–
Severe esophageal varices	1.719 (1.107, 2.697)	0.017	1.329 (0.737, 2.403)	0.345
Gastric Varices	1.970 (1.300, 2.997)	0.001	1.583 (0.918, 2.748)	0.100
Ascites severity				
Mild versus no	1.303 (0.704, 2.399)	0.396	–	–
Moderate/Severe versus no	0.963 (0.530, 1.737)	0.902	–	–
Extension to SV	1.822 (0.978, 3.551)	0.066	0.928 (0.441, 2.001)	0.846
Extension to SMV	2.280 (1.369, 3.901)	0.002	2.194 (1.218, 4.060)	**0.010**
AST (U/L)	0.988 (0.979–0.995)	0.002	0.995 (0.984, 1.001)	0.287
GGT(U/L)	0.996 (0.992, 0.999)	0.027	0.998 (0.994, 1.002)	0.333
TBA (µmol/L)	0.994 (0.989, 0.998)	0.003	0.998 (0.993, 1.002)	0.381
LYMPH (× 10^9^/L)	0.528 (0.306, 0.895)	0.019	0.821 (0.436, 1.577)	0.547
RBC (× 10^12^/L)	0.492 (0.359, 0.666)	< 0.001	1.441 (0.639, 3.219)	0.372
HGB (g/L)	0.967 (0.957, 0.976)	< 0.001	0.967 (0.918, 1.016)	0.183
HCT	0.889 (0.856, 0.921)	< 0.001	0.977 (0.778, 1.239)	0.845

Boldface values indicate statistically significant results (*P* < 0.05)

Analysis of thrombus distribution patterns indicated that thrombus extension into the SMV was significantly associated with an increased risk of bleeding (*P* = 0.002), whereas thrombus extension into the SV did not exhibit a statistically significant association with bleeding risk (*P* = 0.066), although there was a trend toward increased bleeding risk.

Multivariate logistic regression analysis identified SMVT extension as the sole independent factor associated with gastrointestinal bleeding (Table 3). Patients with SMVT extension were significantly associated with an elevated risk of gastrointestinal bleeding compared to those without such extension (OR: 2.194; 95% CI: 1.218–4.060; *P* = 0.01). Conversely, SV thrombus extension was not significantly associated with gastrointestinal bleeding after multivariate adjustment.

### Risk factors for gastrointestinal bleeding in patients with PVT over a 12-month follow-up

Over the 12-month follow-up period, six patients underwent TIPS procedures, five died, and seven were lost to follow-up before reaching endpoints related to gastrointestinal bleeding. The final analysis included 362 patients for whom complete follow-up data were available. Participants were categorized based on the incidence of gastrointestinal bleeding during the 12-month period: 201 patients (55.5%) were classified into the bleeding group, and 161 patients (44.5%) were included in the non-bleeding group. A comparative analysis of baseline data revealed a statistically significant difference between the groups only in terms of SMVT extension (*P* = 0.006), as presented in Table 4.

**Table 4:** Baseline Demographic, Biochemical, and Clinical Characteristics of patients with gastrointestinal bleeding at 12 months of follow-up

Variables	Non-bleeding Group (*n* = 161)	Bleeding Group (*n* = 201)	*P*-value
Demographics			
Male gender, n (%)	61 (37.9)	72 (35.8)	0.685
Age (years)	56.74 ± 11.28	57.16 ± 10.66	0.718
Etiology of cirrhosis, n (%)			0.823
- Viral	85 (52.8)	110 (54.7)	
- Alcohol	28 (17.4)	37 (18.4)	
- Others	48 (29.8)	54 (26.9)	
Child-Pugh Class, n (%)			0.723
- A	26 (16.1)	39 (19.4)	
- B	113 (70.2)	135 (67.2)	
- C	22 (13.7)	27 (13.4)	
Laboratory Parameters			
MELD score	7.10 (6.00, 9.85)	7.01 (6.00, 10.23)	0.921
AST (U/L)	31.00 (24.00, 43.00)	31.00 (24.00, 42.00)	0.745
ALP (U/L)	92.00 (69.00, 123.00)	90.00 (69.00, 130.00)	0.526
GGT (U/L)	30.00 (20.00, 59.00)	32.00 (19.00, 60.00)	0.685
TBIL (µmol/L)	25.80 (16.60, 37.00)	22.10 (15.40, 36.10)	0.210
TBA (µmol/L)	34.00 (15.00, 68.00)	30.00 (13.00, 55.00)	0.226
GLU (mmol/L)	5.20 (4.60, 6.40)	5.30 (4.70, 7.00)	0.170
CRE (µmol/L)	70.00 (62.00, 86.00)	73.00 (66.00, 87.00)	0.131
LYMPH (× 10^9^/L)	0.67 (0.48, 0.95)	0.65 (0.41, 0.80)	0.820
RBC (× 10^12^/L)	3.42 ± 0.71	3.38 ± 0.73	0.629
HGB (g/L)	101.94 ± 24.94	99.06 ± 25.48	0.280
HCT	31.28 ± 6.62	30.50 ± 6.83	0.275
PLT (× 10^9^/L)	59.00 (40.00, 83.00)	60.00 (42.00, 83.00)	0.677
Complications			
Encephalopathy, n (%)	8 (5.0)	18 (9.0)	0.144
Ascites severity, n (%)			0.468
- None	22 (13.7)	36 (17.9)	
- Mild (< 3 cm)	66 (41.0)	73 (36.3)	
- Moderate/Severe (≥ 3 cm)	73 (45.3)	92 (45.8)	
Severe esophageal varices, n (%)	51 (31.7)	71 (35.3)	0.466
Gastric Varices, n (%)	90 (55.9)	121 (60.2)	0.410
Thrombus Characteristics			
Thrombus non-extensions, n (%)			0.060
- Type 1 (MPV)	65 (52.0)	55 (42.3)	
- Type 2 (PV branch)	21 (16.8)	16 (12.3)	
- Type 3 (MPV + PV branch)	39 (31.2)	59 (45.4)	
Thrombus Extensions, n (%)			
- SV	17 (10.6)	34 (16.9)	0.084
- SMV	26 (16.1)	57 (28.4)	**0.006**
Portal Cavernoma, n (%)	22 (13.7)	35 (17.4)	0.331
Portosystemic Shunt, n (%)	39 (24.2)	60 (29.9)	0.233

Boldface values indicate statistically significant results (*P* < 0.05)

A univariate logistic regression analysis was conducted to evaluate 37 variables, identifying those with a significance level of *P* < 0.1, specifically thrombus extension to the SMV, ALP, and TBIL, for inclusion in a multivariate logistic regression model. The findings indicated that only thrombus extension to the SMV (*P* = 0.018) and ALP level (*P* = 0.006) were independent factors associated with gastrointestinal bleeding within the 12-month period. Notably, although SV thrombus extension was associated with an increased risk of bleeding in the univariate analysis (*P* = 0.087), multivariate analysis did not demonstrate a significant correlation with gastrointestinal bleeding (Table 5).

**Table 5:** Univariate and Multivariable Logistic Regression Analyses of Factors Associated with Gastrointestinal Bleeding in Patients at 12 months of follow-up

Variable	Univariate Analysis		Multivariable Analysis	
	OR (95% CI)	*P*-value	OR (95% CI)	*P*-value
Sex (Female versus Male)	0.915 (0.595, 1.407)	0.685	–	–
Age	1.004 (0.985, 1.023)	0.716	–	–
Etiology of cirrhosis			–	–
Viral	Reference	–	–	–
Alcohol	1.021 (0.581, 1.810)	0.942	–	–
Others	0.869 (0.537, 1.408)	0.568	–	–
Child-Pugh class			–	–
B versus A	0.796 (0.453, 1.382)	0.422	–	–
C versus A	0.818 (0.385, 1.736)	0.600	–	–
MELD score	0.997 (0.943, 1.055)	0.921	–	–
Severe esophageal varices	1.178 (0.760, 1.835)	0.466	–	–
Gastric Varices	1.193 (0.784, 1.817)	0.410	–	–
Ascites severity				
Mild versus no	0.676 (0.358, 1.257)	0.220	–	–
Moderate/Severe versus no	0.770 (0.413, 1.413)	0.404	–	–
Extension to SV	1.725 (0.937, 3.282)	0.087	1.356 (0.691, 2.722)	0.382
Extension to SMV	2.055 (1.233, 3.500)	0.007	1.962 (1.131, 3.468)	**0.018**
ALP (U/L)	1.003 (1.000, 1.006)	0.087	1.006 (1.002, 1.011)	**0.006**
TBIL (µmol/L)	0.992 (0.983, 1.001)	0.074	0.992 (0.981, 1.002)	0.137

Boldface values indicate statistically significant results (*P* < 0.05)

### Relationship between the extension of SMVT and the risk of gastrointestinal bleeding

This study conducted a comprehensive analysis of the baseline characteristics of 89 patients with thrombus extension to the SMVT and 291 patients without such extensions. Detailed data are presented in Supplementary Table S1. To mitigate the influence of confounding variables, PSM was performed using a 1:2 ratio. Variables were selected based on dual criteria: association with gastrointestinal bleeding (*P* < 0.1 in univariate analysis) and intergroup baseline differences (SMD > 0.2). The included confounding variables were severe esophageal varices, gastric fundus varices, SV extension, and laboratory parameters such as AST, GGT, TBA, GLU, LYMPH, RBC, HGB, and HCT.

The results of the baseline-adjusted comparisons between the two groups, both before and after PSM, are shown in Table 6, and the SMD diagram for clamp matching is displayed in Supplementary Figure S1. These findings indicate a satisfactory post-matching intergroup balance. After PSM, a final analysis was performed, involving 75 patients with SMVT and 141 patients without SMVT. Statistical analyses revealed significant differences between the two groups in two outcome indicators: History of gastrointestinal bleeding at baseline enrollment (*P* = 0.035) and incidence of gastrointestinal bleeding during the 12-month follow-up period (*P* = 0.033) (Supplementary Table S2).

**Table 6:** Baseline Characteristics of Patients with and without SMVT after PSM

Variables	Total (*n* = 216)	Non-SMVT Group (*n* = 141)	SMVT Group (*n* = 75)	*P*-value
Severe esophageal varices, n (%)	65 (30.1)	44 (31.2)	21 (28.0)	0.625
Gastric varices, n (%)	118 (54.6)	77 (54.6)	41 (54.7)	0.994
SV extensions, n (%)	31 (14.4)	18 (12.8)	13 (17.3)	0.362
AST (U/L)	29.00 (22.75, 36.00)	28.00 (22.00, 36.00)	30.00 (24.00, 36.50)	0.337
GGT (U/L)	30.00 (19.00, 54.25)	29.00 (19.00, 49.00)	31.00 (21.00, 58.00)	0.233
TBA (µmol/L)	24.50 (10.00, 42.00)	24.00 (10.00, 42.00)	25.00 (9.00, 45.50)	0.870
GLU (mmol/L)	5.30 (4.70, 7.23)	5.20 (4.60, 7.20)	5.50 (4.80, 7.50)	0.352
LYMPH (× 10^9^/L)	0.59 (0.40, 0.77)	0.60 (0.40, 0.76)	0.57 (0.40, 0.78)	0.750
RBC (× 10^12^/L)	3.40 ± 0.68	3.42 ± 0.72	3.37 ± 0.60	0.594

## Discussion

The cohort of cirrhotic patients with PVT established in this study exhibited two notable characteristics. First, it comprised 380 patients with cirrhosis, thereby achieving a significantly larger sample size compared to previous studies [12, 13]. Second, by meticulously excluding patients with cancer and those who had undergone splenectomy, splenic embolization, TIPS, or anticoagulation therapy, this study effectively eliminated potential confounding factors that could influence the formation and progression of PVT. This methodological approach ensures homogeneity within the retrospective cohort, thereby providing a robust foundation for precise investigation of disease associations.

First, this study characterized the distribution patterns of PVT in patients with cirrhosis. Our findings indicate that 87.1% of patients exhibited thrombi involving the main trunk of the portal vein, underscoring its role as both the primary origin and central site for PVT formation in cirrhosis. Analysis of anatomical extension patterns revealed that cases with isolated branch involvement (10.3%) or isolated extrahepatic SV/SMV involvement (1.1%) were exceedingly rare. This distribution pattern demonstrates the progression pathway of PVT in cirrhosis, wherein thrombi typically progressively extend from the portal vein trunk to the intrahepatic or extrahepatic veins. This progression contrasts significantly with the localized characteristics of residual SV, which is prone to thrombosis after splenectomy [14]. Furthermore, it differs from the distribution patterns of hepatic cancer emboli [15], which tend to infiltrate intrahepatic branches, suggesting that PVT pathogenesis varies heterogeneously depending on the underlying etiology.

Second, this study demonstrated the practicality and simplicity of classifying PVT based on its anatomical location and extent. Utilizing Sarin’s classification method [11], we categorized PVT into non-extending and extending types based on SV/SMV involvement. The findings revealed that extension to the SV or SMV is relatively uncommon, potentially indicating a stage-specific disease progression or particular clinical implications. Notably, there is a correlation between extended PVT and elevated bleeding risk, which is consistent with the pathophysiological perspective that extrahepatic venous extension may be associated with exacerbated portal hypertension and increased variceal fragility. This supports the utility of anatomical classification in identifying high-risk patients, as thrombus extension to the SV or SMV denotes a more severe hemodynamic disturbance that is associated with an increased likelihood of variceal rupture.

PVT demonstrates considerable heterogeneity in terms of anatomical characteristics, host-related factors, and disease manifestations, posing a challenge for the development of a classification system that can adequately address individual patient variability [6]. This heterogeneity reduces the clinical utility of existing classification systems and impedes the formulation of standardized decision-making criteria. For instance, the Yerdel classification is instrumental in guiding vascular reconstruction planning during liver transplantation, whereas the MAROT classification is more appropriate for interventional therapies such as TIPS [16]. The novel classification method proposed by Sarin [11] transcends traditional anatomical frameworks by integrating further therapeutic management-related information, including thrombus extent, occlusion severity, disease duration, clinical symptoms, and underlying liver disease types. This comprehensive approach facilitates thorough evaluation and treatment planning for thrombosis. However, the inclusion of numerous factors may limit their clinical applicability. In contrast, our classification protocol, which is based on anatomical location and lesion extent, provides operational simplicity while delivering clear prognostic value. Clinicians can efficiently implement this classification using standard imaging modalities, such as ultrasound and contrast-enhanced CT or MRI, thereby facilitating accurate stratification of gastrointestinal bleeding risks.

Third, this study offers preliminary insights into the clinical relevance of classifying and management. Current guidelines typically recommend an observational approach for asymptomatic PVT patients who are not transplant candidates, provided that the thrombosis does not involve SMV and the portal vein main trunk obstruction is < 50% [17–19]. However, our findings indicated that 87.1% of the patients demonstrated involvement of the main trunk. This high prevalence suggests that relying solely on the degree of main trunk occlusion in clinical practice may result in delayed treatment in many patients owing to potential discrepancies in imaging assessments. Notably, our research demonstrates that thrombus extension to the SMV or SV is significantly associated with an increased risk of gastrointestinal bleeding compared with cases without such an extension. This underscores the necessity for more aggressive interventions in these patients, particularly in optimizing the timing of therapy in 29.1% of cases with this extension, which is of considerable clinical importance.

Fourth, this study identified SMVT extension is closely associated with gastrointestinal bleeding in patients with cirrhosis and PVT, and was identified as an independent associated factor. Specifically, multivariate logistic regression analysis revealed that patients with SMVT extension had a 2.194-fold increased risk of baseline gastrointestinal bleeding compared to those without such extension. During the follow-up period, the results of the Kaplan-Meier curve analysis further corroborate that bleeding events in patients with SMVT positivity were predominantly concentrated within the initial six months. This temporal distribution characteristic offers a more precise foundation for clinical risk stratification.

Previous research has indicated that SMVT can result in acute mesenteric ischemia in 4%-58% of cases, with mortality rates ranging from 7% to 45% [20–23]. Consequently, anticoagulation therapy is recommended for patients with SMVT, with some studies advocating for lifelong treatment [17–19]. Notably, however, these findings are predominantly derived from studies involving patients with acute and subacute SMVT [20–23]. These patients frequently encounter difficulties in developing adequate venous collateral circulation to promptly re-establish intestinal blood flow, often presenting with abdominal pain and other symptoms indicative of intestinal ischemia. Therefore, anticoagulation therapy is considered an essential therapeutic intervention.

Conversely, SMVT in patients with cirrhosis generally manifests as a chronic, asymptomatic condition, frequently identified incidentally during imaging studies [24]. Patients with chronic non-obstructive SMVT develop collateral circulation, which provides alternative venous drainage pathways and delays the onset of ischemic symptoms partially. Our study also demonstrated that among 89 patients with SMVT, only one experienced intestinal ischemic necrosis, with 98.9% remaining asymptomatic. Nonetheless, the clinical significance, prognosis, and therapeutic requirements of patients with chronic asymptomatic SMVT have not been thoroughly investigated.

A recent retrospective study suggested that untreated asymptomatic SMVT does not substantially increase the risk of mortality or hepatic decompensation in patients with cirrhosis. Consequently, the study discourages using potentially hazardous prophylactic anticoagulation therapy and instead recommends vigilant monitoring [25]. Our research corroborates that SMVT is not only independently associated with gastrointestinal bleeding but also significantly associated with an increased risk of rebleeding. Zhang et al. [26] further identified SMVT as an independent risk factor for postoperative rebleeding occurring six weeks after esophageal variceal ligation in patients with PVT following gastrointestinal bleeding. They posited that SMVT impairs hepatic blood flow, resulting in intestinal edema, bacterial translocation, and liver dysfunction, which collectively elevate rebleeding rates, a finding that aligns with our conclusions. Consequently, early intervention in such patients is of substantial clinical importance in preventing bleeding, which is the primary outcome of this study.

Simultaneously, it is important to consider that the extension of SMVT may not independently drive bleeding but could instead serve as a surrogate marker for advanced or severe portal hypertension. Our findings are closely aligned with current national and international guidelines. Specifically, the Baveno VII Consensus explicitly states that anticoagulation “should be considered” when thrombosis involves the SMV [19]. Our results further validate the rationale for classifying SMVT as a high-risk category in these guidelines, providing empirical support from a clinical cohort to reinforce the guideline recommendations.

However, this also reveals a key clinical management dilemma: the “aggressive intervention” for patients with SMVT extension may not merely involve optimizing the timing of anticoagulation, but rather reducing bleeding risk through standardized prophylactic treatment of esophagogastric varices to enable safe anticoagulation. Our study revealed that only 8 patients (2.1%) received anticoagulant therapy during follow-up, indicating that most were not treated due to clinicians’ concerns about bleeding risks. Among those who did receive anticoagulation, a 100% bleeding rate in both the baseline non-bleeding and SMVT extension groups underscored the potential risks. Therefore, the core pathway of “aggressive intervention” for patients with SMVT extension should be “prophylactic variceal management + safe anticoagulation”: first, standardize the prophylactic management of variceal risk via endoscopic therapy or NSBBs, and initiate anticoagulation only after the acute bleeding risk is reduced to a controllable range, achieving the dual goals of thrombosis prevention and bleeding control.

For complex cases with uncontrollable portal hypertension or contraindications to anticoagulation, TIPS is an important alternative intervention. The study by Luo et al. [27] provides key evidence-based support for this: their study on patients with chronic portal vein occlusion showed that TIPS has a technical success rate of 94.3%, and adequate inflow from the SMV or SV (maximum diameter ≥ 8 mm) is a core predictor of long-term shunt patency. This is highly consistent with the core anatomical feature of “SMV involvement” emphasized in our study, indicating that vascular inflow status, which is closely related to thrombus extension patterns, may also serve as an important consideration in treatment decision-making for cirrhotic patients with PVT.

This study underscores that SMVT extension is strongly and independently associated with gastrointestinal bleeding, serving as a key indicator for risk stratification. However, the clinical implications of the SV thrombus extension warrant careful consideration. Anatomically, the SMV collects venous blood from the small intestine and right colon, and then drains into the main portal vein, which serves as its primary blood conduit [28]. Conversely, the SV predominantly drains blood from the spleen, with minor contributions from the short gastric vein and left gastroepiploic vein. Consequently, thrombosis in the SV increases venous pressure in the spleen and gastric fundus regions, potentially leading to the formation of varices [29].

Clinical data suggest that although gastrointestinal bleeding resulting from ruptured gastric varices constitutes a relatively minor proportion of cases, it poses substantial challenges in achieving hemostasis and is associated with high mortality rates [30, 31]. Our study revealed a significantly higher incidence of gastric varices in the bleeding group, compared to the non-bleeding group, thereby reinforcing this pathophysiological mechanism. Notably, our study observed a trend toward increased bleeding risk in patients with SV extension, this did not reach statistical significance in multivariate analysis, potentially attributed to the limited sample size (51 patients, 13%) of SV-involved cases and concurrent SMV extension in some patients. Nevertheless, the higher prevalence of gastric varices in bleeding patients highlights the clinical relevance of SV involvement in assessing variceal bleeding risk, warranting cautious surveillance in this subgroup of cirrhotic PVT patients.

This study identified elevated ALP as an independent risk factor for gastrointestinal bleeding within a 12-month period. However, current evidence does not clearly establish a direct link between ALP and either portal hypertension or gastrointestinal bleeding. The potential association may be attributed to intrahepatic cholestasis and progressive fibrosis, as indicated by elevated ALP levels, which could indirectly exacerbate portal hypertension [32]. Despite the statistical significance of this finding, the OR is close to 1, suggesting that its utility may be limited to providing supplementary information for bleeding risk stratification at the statistical level. Further research is necessary to elucidate the precise mechanism of action and clinical relevance of this association.

This research, characterized as a large-sample retrospective study, meticulously excluded confounding variables and applied PSM to effectively control biases, providing robust data to demonstrate the PVT classification and the association between SMVT extension and portal hypertension-related gastrointestinal bleeding.

However, the retrospective design still has inherent limitations like selection bias. A key limitation is temporal ambiguity regarding the sequence of baseline bleeding and SMVT extension. Consequently, our findings indicate association rather than causation. Another limitation is that bleeding was only considered as esophagogastric variceal rupture, excluding lower gastrointestinal bleeding from ectopic varices or portal hypertensive enteropathy. Although no relevant bleeding was recorded, retrospective documentation bias might underestimate the overall bleeding risk in patients with SMVT extension. Furthermore, the absence of direct hemodynamic indicators, such as portal venous flow velocity and hepatic venous pressure gradient (HVPG), limits the ability of Child-Pugh and MELD scores to accurately assess the severity of portal hypertension. Consequently, this limitation hinders our capacity to definitively ascertain whether the extension of SMVT is an independent contributor to bleeding or merely serves as a surrogate marker for advanced or severe portal hypertension. Furthermore, while PSM has successfully balanced the key confounding factors included in the analysis, there remain unmeasured potential confounders, such as the frequency of prior bleeding, anticoagulant therapy, and thrombus-related imaging details, among others. Although these potential confounders may not have substantially compromised the robustness of the primary conclusion, they necessitate further control in subsequent research endeavors.

Therefore, future research should aim to validate these findings in larger, multicenter prospective cohorts to enhance robustness and external validity. Future prospective studies that incorporate direct hemodynamic measurements like HVPG will be crucial to precisely quantify and disentangle the independent contributions of various risk factors (including variceal severity and thrombus anatomy) to the bleeding risk in cirrhotic patients with PVT. Additionally, studies incorporating dynamic imaging to monitor thrombus progression could provide deeper insights into the temporal relationship between SMVT extension and bleeding events. Finally, interventional trials are warranted to investigate whether proactive anticoagulation in cirrhotic patients with SMVT can effectively reduce the incidence of gastrointestinal bleeding, thereby informing evidence-based clinical guidelines.

## Supplementary Information

Supplementary Material 1.

## Data Availability

The data that support the findings of this study are available on request from the corresponding author. The data are not publicly available due to privacy or ethical restrictions.

## Abbreviations

PVT: Portal vein thrombosis
MELD: Model for end-stage liver disease
NSBBs: Non-selective beta-blockers
TIPS: Transjugular intrahepatic portosystemic shunt
MPV: Main trunk of the portal vein
PV: Portal vein
SV: Splenic vein
SMV: Superior mesenteric vein
SMVT: Superior mesenteric vein thrombosis
PSM: Propensity score matching
SMD: Standardized mean difference
MASLD: Metabolic-associated steatotic liver disease
HVPG: Hepatic venous pressure gradient
