# Supplementary material for: Gastrointestinal bleeding risk in cirrhotic portal vein thrombosis: focus on thrombus extension to superior mesenteric vein
Source: BMC Gastroenterol. 2026 Mar 3;26:209. doi: 10.1186/s12876-026-04710-y (PMC13064282; doi:10.1186/s12876-026-04710-y)
Supplement: Supplementary file 1 — Supplementary Material 1. [file 12876_2026_4710_MOESM1_ESM.docx]

**Supplementary Table S1.** Baseline Demographic, Biochemical, and Clinical Characteristics of patients according to the presence or absence of SMVT.

| **Variables** | **Non-SMVT Group**  **(n = 291)** | **SMVT Group**  **(n = 89)** | ***P-*value** |
| --- | --- | --- | --- |
| **Demographics** |  |  |  |
| Male gender, n (%) | 177 (60.8) | 63 (70.8) | 0.088 |
| Age (years) | 57.36 ± 11.04 | 56.00 ± 10.84 | 0.302 |
| **Etiology of cirrhosis, n (%)** |  |  | 0.142 |
| - Viral | 144 (49.5) | 54 (60.7) |  |
| - Alcohol | 57 (19.6) | 16 (18.0) |  |
| - Others | 90 (30.9) | 19 (21.3) |  |
| **Child-Pugh Class, n (%)** |  |  | 0.704 |
| - A | 56 (19.2) | 14 (15.7) |  |
| - B | 193 (66.3) | 63 (70.8) |  |
| - C | 42 (14.4) | 12 (13.5) |  |
| **Laboratory Parameters** |  |  |  |
| MELD score | 6.80 (6.00, 9.82) | 7.25 (6.00, 10.85) | 0.371 |
| AST (U/L) | 32.00 (25.00, 45.50) | 30.00 (23.00, 37.00) | **0.017** |
| ALP (U/L) | 93.00 (71.00, 129.00) | 86.00(65.00, 120.00) | 0.095 |
| GGT (U/L) | 31.00 (19.00, 60.50) | 31.00 (20.00, 56.00) | 0.865 |
| TBIL (µmol/L) | 22.40 (15.95, 37.20) | 23.60 (16.50, 35.50) | 0.967 |
| TBA (µmol/L) | 35.00 (14.00, 68.00) | 24.00 (9.00, 41.00) | **0.002** |
| GLU (mmol/L) | 5.20 (4.60, 6.50) | 5.50 (5.00, 7.60) | **0.010** |
| CRE (µmol/L) | 72.00 (63.00, 86.50) | 76.00 (66.00, 87.00) | 0.160 |
| LYMPH (× 10^9^/L) | 0.61 (0.44, 0.90) | 0.56 (0.39, 0.78) | 0.051 |
| RBC (× 10^12^/L) | 3.37 ± 0.72 | 3.38 ± 0.69 | 0.870 |
| HGB (g/L) | 99.85 ± 24.73 | 98.37 ± 25.32 | 0.628 |
| HCT | 30.82 ± 6.64 | 30.28 ± 6.86 | 0.514 |
| PLT (× 10^9^/L) | 61.00 (42.00, 84.50) | 56.00 (41.00, 74.00) | 0.212 |
| **Complications** |  |  |  |
| Encephalopathy, n (%) | 23 (7.9) | 9 (10.1) | 0.511 |
| Ascites severity, n (%) |  |  | 0.404 |
| - None | 50 (17.2) | 10 (11.2) |  |
| - Mild (< 3 cm) | 110 (37.8) | 36 (40.4) |  |
| - Moderate/Severe (≥ 3 cm) | 131 (45.0) | 43 (48.3) |  |
| Severe esophageal varices, n (%) | 103 (35.4) | 25 (28.1) | 0.202 |
| Gastric Varices, n (%) | 171 (58.8) | 52 (58.4) | 0.932 |
| Portal Cavernoma, n (%) | 35 (12.0) | 35 (12.0) | **< 0.001** |
| Portosystemic Shunt, n (%) | 74 (25.4) | 30 (33.7) | 0.125 |
| History of gastrointestinal bleeding, n (%) | 158 (54.3) | 65 (73.0) | **0.002** |
| Gastrointestinal bleeding within 12 months, n (%) | 146 (50.2) | 60 (67.4) | **0.004** |

**Supplementary Table S2.** Comparison of Outcomes Between Groups with and without SMVT after PSM.

| **Variables** | **Category** | **Total**  **(n = 216)** | **Non- SMVT Group (n = 141)** | **SMVT Group (n = 75)** | ***P*-value** |
| --- | --- | --- | --- | --- | --- |
| **History of gastrointestinal bleeding, n (%)** | No | 81 (37.5) | 60 (42.6) | 21 (28.0) | **0.035** |
|  | Yes | 135 (62.5) | 81 (57.4) | 54 (72.0) |  |
| **Gastrointestinal bleeding within 12 months, n (%)** | No | 90 (43.7) | 67 (48.9) | 23 (33.3) | **0.033** |
|  | Yes | 116 (56.3) | 70 (51.1) | 46 (66.7) |  |

**Supplementary Figure**


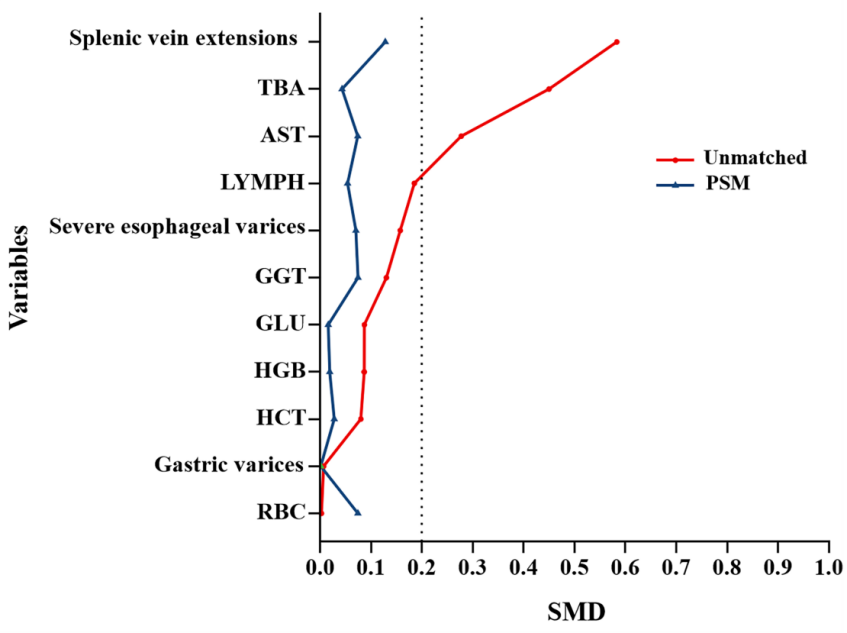


**Supplementary Figure S1.** SMD diagram of PSM in patients with or without SMVT extension.
